# Overexpression of *1-deoxy-D-xylulose-5-phosphate reductoisomerase* enhances the monoterpene content in *Litsea cubeba*

**DOI:** 10.48130/FR-2023-0011

**Published:** 2023-04-24

**Authors:** Yunxiao Zhao, Yingguan Liu, Yicun Chen, Ming Gao, Liwen Wu, Yangdong Wang

**Affiliations:** 1 State Key Laboratory of Tree Genetics and Breeding, Chinese Academy of Forestry, Beijing 100091, China; 2 Research Institute of Subtropical Forestry, Chinese Academy of Forestry, Hangzhou 311400, China

**Keywords:** *Litsea cubeba*, Monoterpene, DXP reductoisomerase, MEP pathway

## Abstract

Monoterpenes are important components of plant essential oils and have long been used as raw materials for spices and food flavorings. *Litsea cubeba* is an economically aromatic plant species, the fruits of which produce essential oil with monoterpenes as the dominant components. As a branch point of carbon flow in the methyl erythritol phosphate (MEP) biosynthesis pathway, 1-deoxy-D-xylo-5-phosphate reductoisomerase (DXR) is a key rate-limiting enzyme that catalyzes the MEP pathway’s second committed step. Therefore, *DXR* has become an effective regulation target to improve the biosynthesis of plant monoterpenes. In this study, we identified a *DXR* from *L. cubeba*, which was highly expressed in fruits, induced by MeJA and repressed by darkness. An enzyme assay showed that recombination LcDXR protein catalyzed *DXP* with NADPH as the cofactor. Transient overexpression of *LcDXR* significantly increased the content of monoterpenes in *L. cubeba*. Furthermore, *LcDXR*-overexpressing tobaccos were conducted and showed almost 5.9-fold increase in monoterpenes production, including limonene, α-pinene, eucalyptol, linalool, terpineol and camphor. Overexpression of *LcDXR* activated the metabolic flux of monoterpene biosynthesis through crosstalk and feedback mechanism. In addition, *LcDXR*-overexpressing tobaccos had no effect on phenotype of transgenic tobaccos. Our results demonstrate that *LcDXR* is a critical regulator of the monoterpene production in *L. cubeba* and other plants.

## Introduction

Plants produce large amounts of natural products with specialized functions, which are crucial to their development, defense responses and the interactions with the ecological environment^[1]^. Among these products, terpenes constitute an important and diverse category and play an important role in primary and secondary metabolism of plants. In primary metabolism, terpenoids such as gibberellin, cytokinin, auxin and carotenoid are widely involved in plant growth and development^[2]^. Defense against biological and abiotic stress is the most important function of terpenoids, which are highly diverse and species-specific as secondary metabolites^[3]^. In human society, terpenes are widely utilized in flavor, perfume, medicine and ecological pesticide^[4−6]^. Although terpenes have considerable economic value, it is still a major challenge to improve their utilization efficiency, especially for wild resources. Monoterpenes, as C_10_ terpenes, typically colorless, lipophilic, volatile, and antibacterial, are the source of the unique fragrance and flavor of essential oils^[7,8]^. Monoterpenes consist of isopentenyl diphosphate (IPP) and dimethylallyl diphosphate (DMAPP), which are isomeric 5-carbon structural units, derived from the mevalonate (MVA) pathway in the cytosol/peroxisome and the methyl erythritol phosphate (MEP) pathway in the plastid^[9]^. Then, one IPP and one DMAPP are catalyzed to form geranyl diphosphate (GPP). The last step is to convert GPP into a monoterpene parent scaffold *via* the monoterpene synthase.

The MEP pathway is mainly controlled by 1-deoxy-d-xylose 5-phosphate synthase (DXS) and 1-deoxy-D-xylulose-5-phosphate reductoisomerase (DXR), which has been known as the rate-limiting enzyme of the MEP pathway in plants^[10,11]^. DXR is an enzyme that catalyzes 1-Deoxy-D-xylulose-5-phosphate to 2-C-Methyl-D-erythritol-4-phosphate, the second step of the MEP pathway^[12]^. It requires the participation of cofactors NADPH, Mn^2+^, Co^2+^, or Mg^2+^ to convert DXP into an important precursor MEP for terpenoid synthesis^[13]^. Since the first *DXR* was cloned from *Escherichia coli*, the gene has been found in several other species and studied for its function in terpenoid synthesis^[14]^. There is transport peptide-guided DXR localization in plastids at the N-terminal, and it has a conservative Cyr-Ser–(Ala/Met/Val/Thr) motif; there is a proline enrichment area P (P/Q) PAWPG (R/T); two DXR functional binding motifs (LPADSEHSAI, NKGLEVIEAHY), and two highly conservative NADPH binding motifs (GSTGS [I/V] GT and LAAGSN [V/I] T)^[15,16]^. Overexpression of key rate-limiting genes in the terpene biosynthesis pathway is an effective way to improve plant terpenoid yield. Interestingly, *DXR* function in terpene biosynthesis seems to be species-specific. Overexpression of the *DXR* effectively promotes monoterpene synthesis of peppermint essential oil, terpene indole alkaloids in *Catharanthus roseus*, artemisinin in *Artemisia annua*, and taxene in transgenic *Arabidopsis thaliana*^[17−21]^. Importantly, *DXR* expression is significantly correlated with the cumulative distribution of monoterpenes^[22−24]^. In contrast, the expression level of *DXR* does not affect the production of terpenes in *Solanum lycopersicum*, *Oryza sativa*, or *Lavandula angustifolia*^[11,25,26]^.

*Litsea cubeba*, belonging to the Lauraceae family, is primary woody oil tree in China^[7]^. *L. cubeba* fruits are rich in essential oil (3%−4%), which is widely used in essence, spices, medicine, cosmetics, industrial raw materials, and other fields^[4,27−29]^. For example, *L. cubeba* essential oil (LcEO) plays an important role in the synthesis of vitamin E/K/a and saponin ketone, which acts as a superior food-flavoring agent^[30]^. Meanwhile, LcEO has broad-spectrum antibacterial activities and contributes to improving the treatment of coronary heart disease and other cardiovascular diseases^[31,32]^. The components of LcEO include terpenes, higher alcohols, and organic acids, depending on the maturity of organs^[4,33]^. Geranial, neral, pinene, linalool, and other monoterpenes make up about 96–98% of the LcEO^[7]^. In our previous study, *LcGPPS.SSU1* and *LcTPS42* were demonstrated as the structure genes involved in the monoterpene biosynthesis pathway, and transcription factor including *ERF* and *MYC2*, effectively improving the production of monoterpenes^[7,34−37]^.

The terpene biosynthesis pathway has been characterized by multi-omics analysis of *L. cubeba*. However, to date, few strategies have been reported to improve the yield of LcEO by regulating the key genes encoding MEP pathways. In this study, we firstly investigated the full-length cDNA and the promoter sequence of *LcDXR*. The expression patterns were analyzed in different tissues and different developmental stages in fruits. Instantaneous overexpression of *LcDXR* in *L. cubeba* was also found to increase monoterpene content. In addition, the enhanced monoterpene biosynthesis capacity was tested by overexpressing *LcDXR* in tobacco. The results indicated that *LcDXR* played a key role in regulating the formation of monoterpenes.

## Materials and methods

### Plant materials

Different tissues of *L. cubeba*, including flowers, leaves, stems, roots, and fruits at different development stages (60, 90, 120, and 150 d after flowering, DAF), were collected from a farm in Hangzhou City, Zhejiang Province, China (30°27′94′′ N, 119°58′43′′ E), then quickly frozen, and stored at −80 °C. For *Agrobacterium*-mediated transformation, *Nicotiana benthamiana* was grown in a growth chamber at 26 °C, with a photoperiod of 16 h/8 h.

### *LcDXR* cloning and sequence analysis

The complete sequence of *LcDXR* was identified based on the *L. cubeba* genome and the DXR conserved domain (PF08436) using HMMER (www.hmmer.org). The full-length amino acid sequences of LcDXR and DXR from other plants were aligned using ClustalW software (www.ebi.ac.uk/Tools/msa/clustalo). Maximum-likelihood phylogenetic tree was constructed based on CIPRES (www.phylo.org) with the JTT model using 1,000 bootstrap replicates.

### RNA extraction and quantitative real-time PCR (qRT-PCR)

To analyze the expression profiles of *LcDXR*, the total RNA of different tissues was extracted and reversed to the first strand cDNA synthetase as previously described^[34]^. For qRT-PCR, *ubiquitin-conjugating (UBC)* gene was used as a reference gene^[38]^. The comparative cycle threshold (Ct) method was used to analyze the expression level of *LcDXR* in different tissues and stages. The primer sequences are listed in Supplemental Table S1. Three biological replications were made for each gene.

### Expression of recombinant LcDXR in *Escherichia coli* and enzymatic assays

To determine the enzyme activity of LcDXR, the full-length CDS of *LcDXR* was inserted into pET28a (+) vector. The recombinant plasmid was transferred to *E. coli* ER2566 (Weidi, China), and the recombinant protein was induced and purified as described previously. The recombinant protein was detected by SDS-PAGE and Western blot analysis.

For *in vitro* enzyme activity assays, 20 μg DXR purified protein was incubated with 50 mM Tris-HCl, 5 mM MgCl_2_, 1.23 mM NADPH, and 0.25M DXP at 30 °C for 15 min and the time course was measured by monitoring the formation of the generated NADPH in a 340 nm spectrophotometer^[14]^. DMSO was added to replace the DXP in the control reaction. Three technological replications were carried out.

### Transient overexpression of *LcDXR* in *L. cubeba*

To investigate the function of *LcDXR* in monoterpene biosynthesis, the transient overexpression of *L. cubeba* was constructed as in a previous study^[34]^. Two-month-old tissue culture seedlings of *L. cubeba* were generated for transient overexpression. The completed CDS of *LcDXR* was cloned into the overexpression vector pCambia1300S. *A. tumefaciens* LBA4404 strains containing empty vector or pCambia1300S*-LcDXR* were cultured and then infiltrated by vacuum into sterility seedling leaves under the same growth condition. The seedlings were then placed on MS medium and cultured at 25–27 °C with a photoperiod of 16 h light/8 h dark for 40–72 h. Leaves were collected for GC-MS and qRT-PCR analysis according to a previous study^[7]^. The target compounds were identified by comparing their mass spectra with those in the NIST08 library.

### Overexpression of *LcDXR* in tobacco

To further confirm the function of *LcDXR*, *pCambia1300S-LcDXR* was transformed into *Agrobacterium* EHA105 strains for tobacco transformation^[39]^. After selection with Hygromycin B and PCR amplification using *LcDXR* specific primer, ten positive *LcDXR* transformation lines were obtained. qRT-PCR was performed to investigate the expression levels of *LcDXR* as described above, and actin was used as the reference gene^[36]^. The effect of introducing *LcDXR* on monoterpene production was determined by GC-MS analysis.

## Results

### Sequence analysis of *LcDXR*

**Figure 1:**
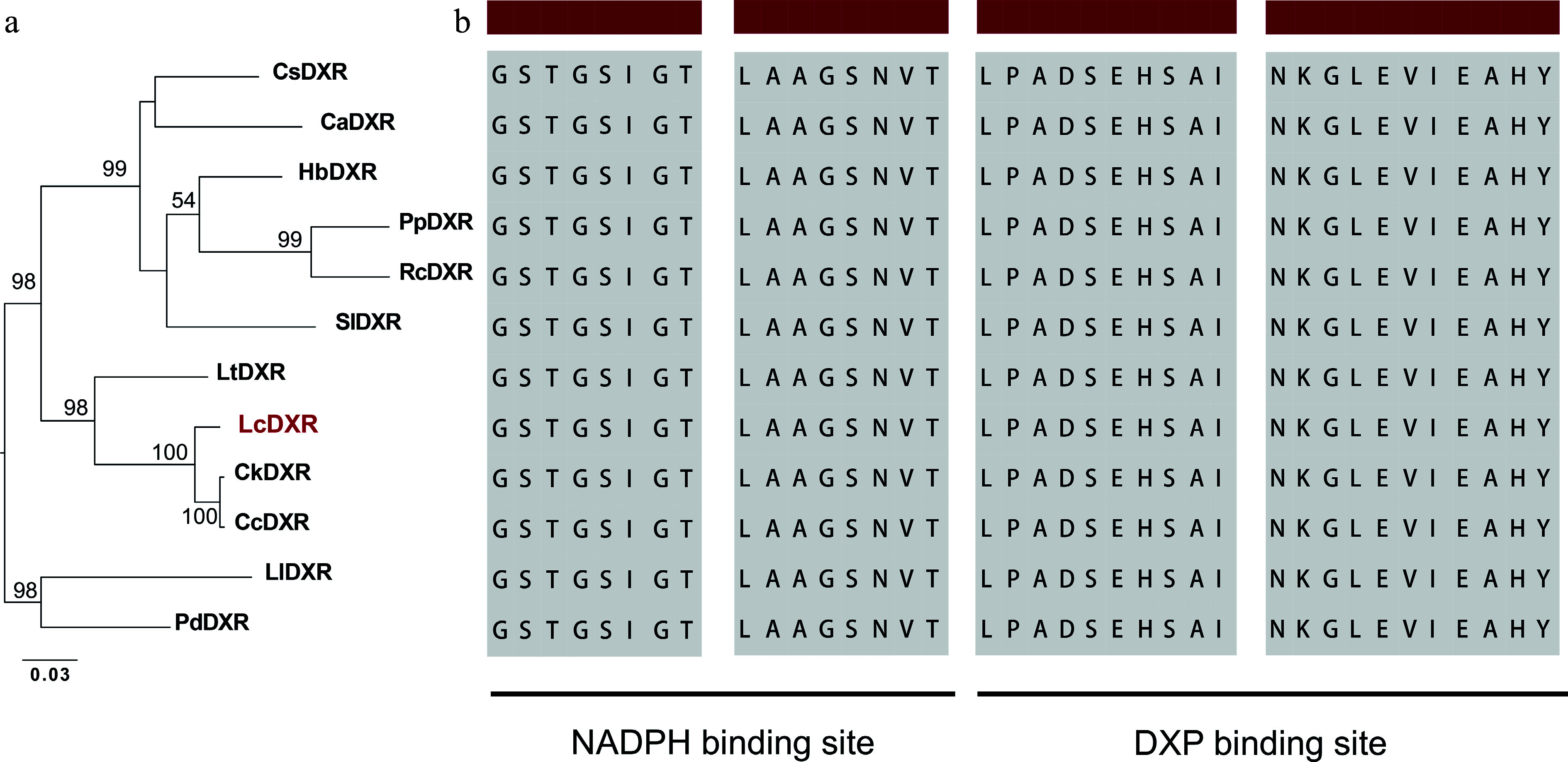
Sequence analysis of the LcDXR. (a) Maximum-likelihood tree of DXR protein sequences, including LcDXR and other DXRs. The following protein sequences were used for the analysis: CsDXR (XP_028055701), CaDXR (XP_027123133), HbDXR (XP_021669849), PpDXR (XP_007210092), RcDXR (XP_024173969), SlDXR (AAK96063), LtDXR (UOO00992), CkDXR (RWR93981), CcDXR (AOW69227), LlDXR (AHJ57307), PdDXR (XP_008787962). (b) Sequence alignment of DXRs. The black background indicates the NADPH binding domain and the DXP binding domain.

The complete *LcDXR* open reading frame was 1,413 bp with a 5' noncoding region of 34 bp and a 3' noncoding region of 53 bp, encoding a protein containing 470 amino acid residues. LcDXR showed high sequence identity (> 85.56%) with previously reported DXRs. In the phylogenetic tree of DXRs, LcDXR grouped near to DXRs from *Cinnamomum kanehirae* and *C. camphora*, which all belong to the Lauaceae family (Fig. 1a). To further investigate sequence variants among DXRs, we conducted multiple sequence alignment of DXR amino acid sequences and found that LcDXR has the conserved domain of LPADSEHSAI and NKGLEVIEAHY for binding DXP and two NADPH binding motifs, GSTGSIGT and LAAGSNVT (Fig. 1b).

### Tissue-specific expression patterns of *LcDXR* in *L. cubeba*

**Figure 2:**
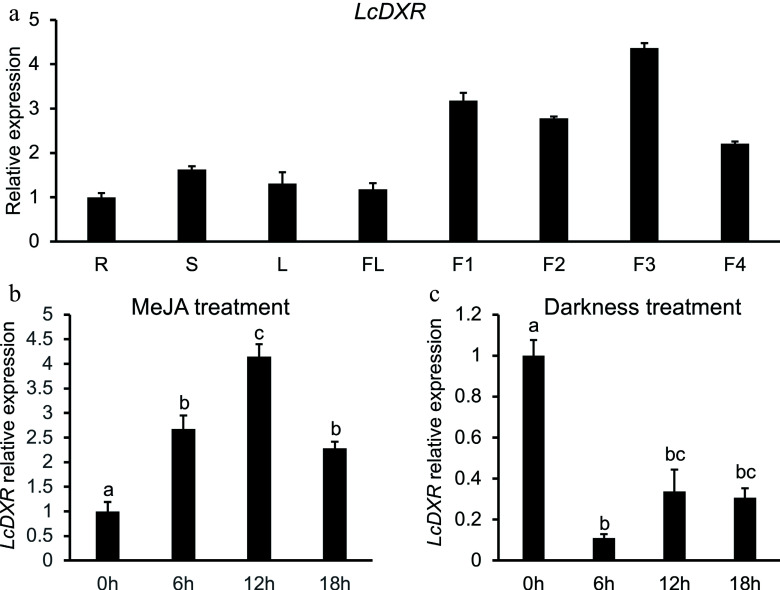
Tissue specific expression patterns of *LcDXR*. (a) The transcript levels of *LcDXR* in different tissues of *L. cubeba*. R: roots; S: stems; L: leaves; FL: flower; F1, fruits at 60 DAF; F2, fruits at 90 DAF; F3, fruits at 120 DAF; F4, fruits at 150 DAF. (b) The expression levels of *LcDXR* were induced by MeJA in 60 DAF fruits. (c) The expression levels of *LcDXR* were repressed by darkness in 60 DAF fruits. Error bars represent ± SD from three biological repeats. The different letters above the bars represent the significant difference (*P* < 0.01).

It has been reported that the production of essential oil increases with the development of fruits in *L. cubeba*^[40,41]^. To study the expression pattern of *LcDXR*, qRT-PCR was performed on the roots, stems, leaves, flowers, and different development stages of fruits of *L. cubeba*. As the results shown in Fig. 2a, *LcDXR* was widely expressed in all tissues and highly expressed during fruit ripening. Interestingly, expression peaks were detected at 60 and 120 DAF of fruits, and the biosynthesis of essential oil reached its peak at the same time^[40]^. These results indicated that *LcDXR* played an important role in primary and secondary metabolism in all tissues of *L. cubeba*. It produced a prominent effect in the accumulation of monoterpenes during the early and middle fruit developmental stages.

In addition, the promoter region (−2,000 bp) of *LcDXR* was amplified and many cis-elements were identified, such as MYB, MYC, G-box, ABRE and other motif; ABA, SA, and MeJA signal transmission were noted (Supplemental Fig. S1). In our previous study, the biosynthesis of terpenes was found to respond to darkness and MeJA treatment^[34,37]^. Corresponding to the peak of monoterpene biosynthesis in *L. cubeba* at 12 h after MeJA treatment, *LcDXR* reached the peak of expression at the same time, which increased 4-fold compared with 0 h (Fig. 2b). In fact, the inhibition of darkness on terpene biosynthesis is involved in energy metabolism, light and other factors. Meanwhile, reduced expression of *LcDXR* was found in *L. cubeba* grown under the dark conditions for 48 h (Fig. 2c). These results confirmed that *LcDXR* was induced by MeJA treatment and repressed by darkness.

### Enzyme activity of *LcDXR*

**Figure 3:**
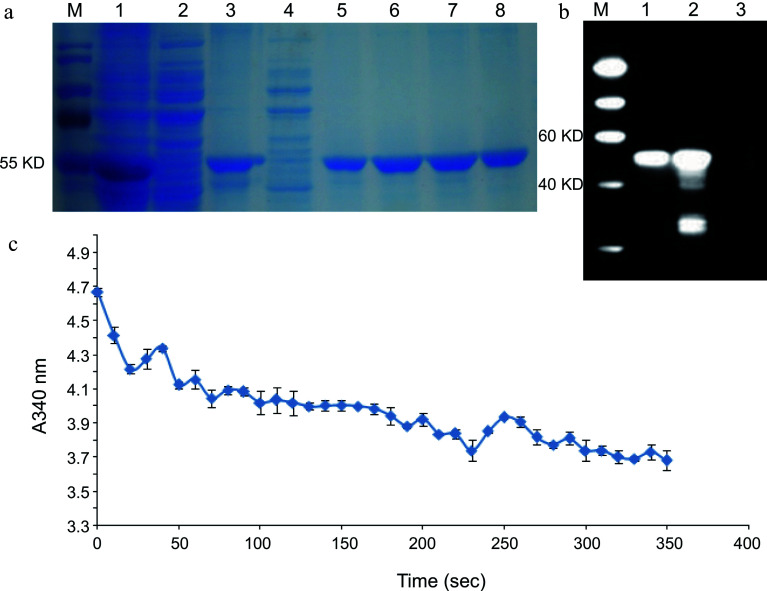
Enzyme activity of LcDXR. (a) SDS-PAGE analysis of recombination protein with His tag. M: Protein Marker, 1: Insoluble protein before purified, 2: Cell lysate supernatant, 3: Transmission fluid, 4: Washing fluid, 5−8: Elution fluid with protein. (b) Western blot analysis of recombinant LcDXR protein. M: Protein Marker, 1: The inclusion protein, 2: Total protein induced in pET–LcDXR2/Arctic Express, 3: Washing fluid. (c) Enzyme activity assay of recombinant LcDXR. NADPH has a robust ultraviolet absorption peak at A340 and NADP+ does not, while the light absorption value of NADPH at A340 decreases during the catalytic reaction. Data represent the mean ± SEs of three biological replicates.

To determine the enzymatic activity of LcDXR, recombinant pET28a plasmid (~63 kDa) with the CDS of *LcDXR* was expressed in *E. coli*. According to SDS-PAGE and western blot analysis, the purified LcDXR recombinant protein had a molecular weight similar to that expected (Fig. 3a, b). The catalytic reaction of DXR requires the conversion of NADPH into NADP+. NADPH has a robust ultraviolet absorption peak at A340 and NADP+ does not, while the light absorption value of NADPH at A340 decreases during the catalytic reaction^[12]^. Accordingly, when LcDXR was added to the enzyme reaction system, NADPH was consumed at A340 rapidly decreased, and the reaction rate slowed in the later stage (Fig. 3c). These results suggested that LcDXR had the catalytic activity.

### Transient overexpression of *LcDXR* increases the terpenes biosynthesis in *L. cubeba*

**Figure 4:**
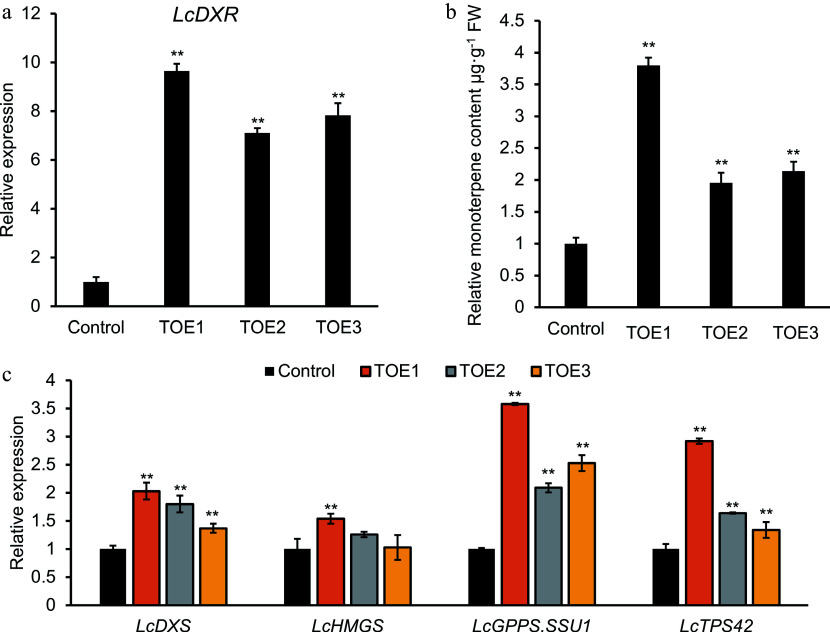
Transient overexpression of *LcDXR* in *L. cubeba* leaves. Leaves were infiltrated with *LcDXR* under the control of the Cauliflower mosaic virus 35S promoter, and three independent transient overexpression lines (TOE) 1-3 were conducted and grown for 2 d, respectively. (a) Relative expression level of *LcDXR* in transient overexpressed *L. cubeba*. (b) The content of monoterpenes in *LcDXR* transient overexpressed *L. cubeba*. (c) Relative expression level of key genes involved in MVA and MEP pathway in *LcDXR* transient overexpressed *L. cubeba*. Data represent the mean ± SEs of three biological replicates. **, *P* < 0.01.

A rapid and efficient transient overexpression system provides a method to detect the gene function in *L. cubeba* without stable transformation system. To investigate whether the overexpression of *LcDXR* contributed to the monoterpene accumulation, transient overexpression analysis was performed in *L. cubeba* leaves and three independent transgenic lines were conducted. Among the *LcDXR* overexpression lines, the expression levels of *LcDXR* were significantly increased by 9.6-, 7.1-, and 7.8-fold compared to controls (Fig. 4a). The introduction of *LcDXR* accelerated the synthesis of monoterpenes, showing an increase of up to 4.0-fold compared with the control (Fig. 4b). It is noteworthy that the *LcDXR* overexpression activated the expression of rate-limiting genes involved in the terpene biosynthesis pathway, including *LcDXS*^[7]^, *LcGPPS.SSU1*^[37]^, and *LcTPS42*^[7]^, which were increased 1.5- to 4.0-fold in overexpressed leaves (Fig. 4c). However, compared to controls, a smaller change seen in 3-hydroxy-3-methylglutaryl CoA synthetase (*LcHMGS*) was investigated, which involved the MVA pathway in the cytoplasm^[2]^. In summary, the transient overexpression of *LcDXR* provided preliminary evidence that *LcDXR* is the key gene for monoterpene biosynthesis of *L. cubeba*.

### Overexpression of *LcDXR* promotes monoterpene biosynthesis in tobacco

**Figure 5:**
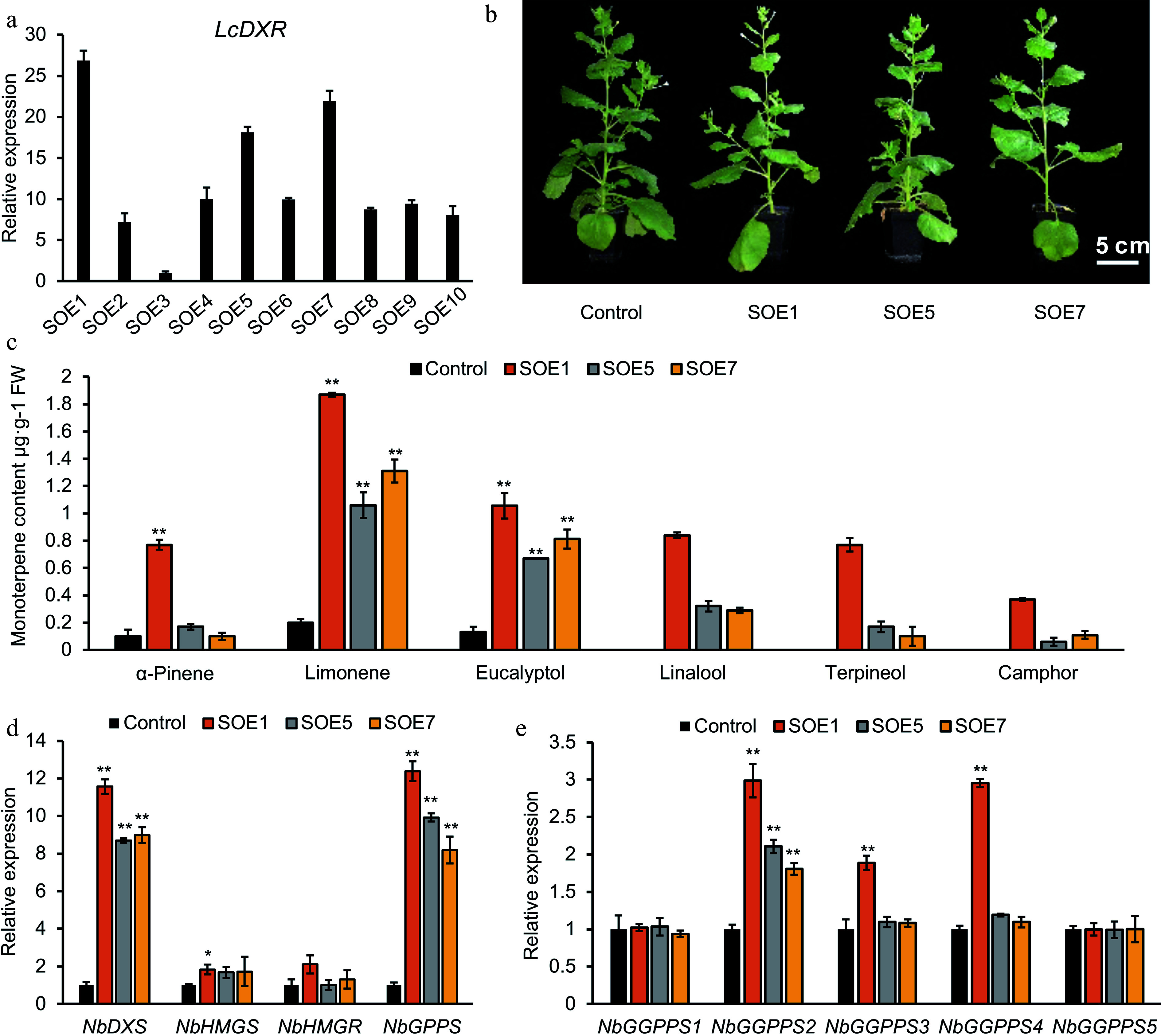
Monoterpene production was increased by overexpression of *LcDXR* in transgenic tobacco. (a) Relative expression of *LcDXR* in ten independent stable overexpression lines (SOE), respectively. (b) The content of monoterpenes in *LcDXR* transgenic tobacco. (c) Different compounds of monoterpenes in transgenic tobacco. (d) Relative expression levels of key genes involved in MVA and MEP pathway. Data represent the mean ± SEs of three biological replicates. *, *P* < 0.05; **, *P* < 0.01.

To further understand the function of *LcDXR*, we constructed transformation tobacco lines carrying *pCambia1300S-LcDXR* vector. Ten independent transgenic lines were identified through hygromycin B selection and PCR amplification. The transcriptional levels of *LcDXR* in stable overexpression lines (SOE) 1, SOE5, and SOE7 were the highest and thus were further used to evaluate monoterpene content and the expression of key genes at the 2-month stage (Fig. 5a). As the result shown in Fig. 5b, *LcDXR* transgenic lines had no obvious morphological changes. The content of monoterpenes collected from leaves was positively correlated with the transcriptional level in different transgenic lines. Compared to the control, the content of limonene, α-pinene, eucalyptol, linalool, terpineol and camphor were significantly increased by *LcDXR* overexpressed. In the leaves of SOE1, SOE5 and SOE7 transgenic tobacco line, limonene was increased from 0.2 μg·g^−1^ towards 1.9 μg·g^−1^ (Fig. 5c). Linalool, terpineol and camphor, which were not detected in the control, showed the content of 0.8 to 0.5 μg·g^−1^ in pCambia1300S-*LcDXR* lines. This result suggested that although tobacco contained many kinds of terpene synthases, the lack of substrates restrict the diversity and yield of monoterpenes.

Furthermore, the expressional level of rate-limiting genes in the MEP pathway encoding *DXS* was upregulated approximately 11-fold in transgenic leaves compared to controls (Fig. 5d). Similarly, as the key gene of the direct precursor for the synthesis of monoterpene, *NbGPPS* was upregulated 12-fold. On the contrary, the expression levels of *NbHMGS* were slightly affected by *LcDXR* through the crosstalk mechanism, and those of *NbHMGR* unchanged. As with monoterpenes, diterpenes are derived from the MEP pathway and located in the chloroplast. Thus, the expression of *GGPPSs*, the direct precursor synthase gene of diterpenoids, were also analyzed. There are five GGPPS genes in tobacco, among which only *NbGGPPS2* significantly increased approximtely 3-fold in all three SOE lines; *NbGGPPS3* and *NbGGPPS4* were only increased in SOE1 (Fig. 5e). In summary, introducing *LcDXR* activated the metabolic flux of the monoterpene biosynthesis pathway.

## Discussion

According to previous studies, precursor supply is a limiting factor for monoterpene biosynthesis through the plastid MEP pathway^[14,20]^. During the process of monoterpene precursor formation in plants, DXR, which catalyzes the conversion of DXP into MEP, is a key rate-limiting enzyme of the MEP pathway^[14]^. IPP and DMAPP synthesized by the MEP pathway mainly contribute to monoterpene biosynthesis. LcEO, containing more than 90% monoterpenoids, has important economic value in commercial and industrial fields. Therefore, *L. cubeba* may be a potentially model plant for studying monoterpene biosynthesis^[7]^. Breaking through the bottleneck of the MEP pathway has been one of the most central challenges for metabolic engineering of monoterpene with the extensive commercial development of plants essential oil^[10]^. Although genes involved in the terpenoid synthesis pathway have been identified in *L. cubeba*, functional analysis to determine the rate-limiting genes is still absent.

Numerous previous studies conducted expression profiling of the *DXR* gene in different plant species and tissues, such as petals and stamens of *R. rugosa* and *Cymbopogon winterianus*, roots of *Salvia miltiorrhiza*, and leaves of peppermint, which have reported a higher level of expression in the main site of isoprenoid biosynthesis^[11,12,14−17,20−26,42]^. In *C. camphora*, *CcDXR* showed the highest expression in flowers, subsequently followed by leaves and stems, respectively^[43]^. In *L. cubeba* fruits, the transcript accumulation of *LcDXR* showed a direct correlation with monoterpene biosynthesis (Fig. 2). In our study, *LcDXR* was expressed in all tissues and up-regulated during fruit development, suggesting that *LcDXR* played a key role in the precursor formation of monoterpenes in fruits, which served as factories for essential oil production, similar to what has been reported in *P. ginseng* and *C. roseus*^[44,45]^. Notably, the highest expression of *LcDXR* was observed at 60 DAF and 120 DAF when the essential oil entered the rapid production phase (Fig. 2). Previous studies have reported that genes related to MEP pathway are highly expressed in photosynthetic tissues^[7]^. In fact, the MEP pathway is located in the chloroplast and responsible for phytohormones, chlorophyll, which is closely related to photosynthesis.

Key enzymes catalyze the synthesis of terpenoid precursors and the formation of various intermediates in the pathway of terpenoid biosynthesis. Thus, regulating the expression of these key enzyme genes to increase the content of downstream target products is an important way for the regulation of terpenoid biosynthesis^[46]^. Increasing the expression of key genes in the metabolic pathway may also affect the metabolic flux of the pathway through feedback regulation or crosstalk mechanism^[47]^. Overexpression of rate-limiting genes involved in the terpene precursor biosynthesis pathway is an effective strategy to improve terpene production, which has been conducted in several plants including *C. roseus*, *W. somnifera*, *A. thaliana*, *Cucumis melo, Solanum lycopersicum, a*nd *Lilium*^[6,10,20,22,44]^. Importantly, DXP is the precursor of not only MEP but also thiamine and pyridoxine^[20]^. As a branch point of carbon flow in the MEP biosynthesis pathway, DXR is a key rate-limiting enzyme in that pathway and an effective target for terpenoid synthesis regulation in plants^[48]^. However, the regulation of the *DXR* gene in plants is species-specific. In particular, in peppermint (Mentha × Piperita), an improvement of 44% in oil yield compared to WT was detected in *DXR* transgenic lines^[21]^. In contrast, overexpression of *DXR* had no effect on lavender^[25]^. Carotenoid accumulation during tomato fruit ripening had no obvious relationship with DXR expression^[11]^. In addition, *SlDXR* did not influence the content of IPP or DMAPP in *Synechococcus leopoliensis*^[49]^. In our study, the concentration of monoterpenes was significantly enhanced in *L. cubeba* leaves overexpressing *LcDXR* compared to the control (Fig. 4). In a previous study, high levels of ectopic expression of *LiDXS* and *LiDXR* induced sclareol biosynthesis without altering growth characteristics and ecological habits^[50]^. It is generally considered that DXS catalyzes the rate-limiting step of the MEP pathway; nevertheless, *DXS*-overexpression-based metabolic engineering strategies are not sufficient to overcome the limitations of utilizing the MEP pathway for high-yield terpene production in tobacco^[47]^. In this study, overexpressing *LcDXR* in tobacco was effective in the activating of genes involved in terpene biosynthesis and leaf monoterpene production (Fig. 5). In fact, although tobacco has the potential to synthesize various monoterpenes, the lack of corresponding substrates limits the monoterpene synthesis in tobacco. Therefore, it is an effective way to improve the terpene content of tobacco by introducing the key genes of highly efficient synthetic substrates through metabolic engineering. It is worth noting that the MEP pathway is not only a precursor pathway for monoterpenes, but also for diterpenoid biosynthesis. Diterpenoids such as chlorophyll and carotenoids are crucial for plant growth and development. Overexpression of designed *NtGGPPS1* in *ntggpps1* mutants increases photosynthetic efficiency and biomass in tobacco leaves^[51]^. However, *NbGGPPS1* was not affected in *LcDXR*-OE lines while *GGPPS2* was significantly increased. In fact, the phenotype was not changed in *LcDXR*-OE lines. In carrots (*Daucus carota*), *DXS* limits the production of chlorophyll in roots and leaves, while *DXR* appears to have a small regulatory effect^[52]^. Obviously, the regulatory effect of MEP pathway on diterpenoids such as chlorophyll, and the synergistic relationship between photosynthesis and terpene synthesis need further research.

The monoterpene synthesis pathway is strong in plasticity through feedback and crosstalk mechanisms^[53−56]^. Previous studies have shown that the increase of downstream products can up-regulate the expression level of upstream genes involved in precursor synthesis pathway through a feedback regulation, to accelerate the metabolic flux of MEP and MVA pathways^[57,58]^ *DXS*, as the first gene of the MEP pathway, is the key target for downstream product feedback regulation of the MEP pathway. In our study, overexpression of *DXR* both promoted the expression of downstream genes by improving the synthesis of precursors and activated the expression of *DXS* genes through the feedback regulation, which together contributed to the increase in monoterpene synthesis Figs 4 & 5). It is worth noting that the key genes of the MVA pathway, *HMGR* and *HMGS*, had smaller change in transgenic *L. cubeba* and tobacco. This result indicated that there was a weaker metabolic flux between the MEP and MVA pathways in *LcDXR* overexpressed lines. The increased metabolic flux in the MEP pathway regulated by *LcDXR* overexpression was not detected in the MVA pathway. In fact, the MEP or MVA pathways tend to preferentially synthesize certain metabolites in different plants^[36]^. Monoterpene, as the main terpenoid (accounting for up to 98% of all terpenoids) in *L. cubeba*, is located in the chloroplast and derived from MEP pathway. In addition, we hypothesize that the contribution of *LcDXR* to monoterpene products is universal, regarding its identical functions to monoterpene biosynthesis in *L. cubeba* and tobacco.

## Conclusions

In conclusion, we functionally characterized *LcDXR*, the key gene of the MEP pathway, which played an important role in the biosynthesis of monoterpenes in *L. cubeba*. Expression analysis suggested that it is the crucial gene to control the production of essential oil monoterpenes in fruits. Enzyme activity assay showed that LcDXR catalyzed DXP with NADPH as a cofactor. Overexpression of *LcDXR* significantly increased the contents of monoterpenes in *L. cubeba* and tobacco. Our results indicated that *LcDXR* could be employed as a universal target gene for metabolism regulation to improve the content of monoterpenes in plant tissues.

## SUPPLEMENTARY DATA

Supplementary data to this article can be found online.

## References

[1] (2019). Exploring the diversity of plant metabolism. Trends in Plant Science.

[2] (1995). Terpenoid metabolism. The Plant Cell.

[3] (2018). Why do plants produce so many terpenoid compounds?. New Phytologist.

[4] (2021). Effects of different frying temperatures on the aroma profiles of fried mountain pepper (*Litsea cubeba* (Lour. ) Pers. ) oils and characterization of their key odorants. Food Chemistry.

[5] (2021). AaMYB15, an R2R3-MYB TF in *Artemisia annua*, acts as a negative regulator of artemisinin biosynthesis. Plant Science.

[6] (2022). SlWRKY35 positively regulates carotenoid biosynthesis by activating the MEP pathway in tomato fruit. New Phytologist.

[7] (2020). The *Litsea* genome and the evolution of the laurel family. Nature Communications.

[8] (2022). The chromosome-scale genome of Phoebe bournei reveals contrasting fates of terpene synthase (TPS)-a and TPS-b subfamilies. Plant Communications.

[9] (2015). Breaking new ground in the regulation of the early steps of plant isoprenoid biosynthesis. Current Opinion in Plant Biology.

[10] (2014). The 2-*C*-methylerythritol 4-phosphate pathway in melon is regulated by specialized isoforms for the first and last steps. Journal of Experimental Botany.

[11] (2001). 1-Deoxy-D-xylulose 5-phosphate reductoisomerase and plastid isoprenoid biosynthesis during tomato fruit ripening. The Plant Journal.

[12] (2013). Evolutionary diversification and characterization of the eubacterial gene family encoding DXR type II, an alternative isoprenoid biosynthetic enzyme. BMC Evolutionary Biology.

[13] (2000). Isolation of the *dxr* gene of *Zymomonas mobilis* and characterization of the 1-deoxy-D-xylulose 5-phosphate reductoisomerase. FEMS Microbiology Letters.

[14] (1998). A 1-deoxy-D-xylulose 5-phosphate reductoisomerase catalyzing the formation of 2-*C*-methyl-D-erythritol 4-phosphate in an alternative nonmevalonate pathway for terpenoid biosynthesis. Proceedings of the National Academy of Sciences of the United States of America.

[15] (2021). In silico characterization and differential expression analysis of 1-deoxy-d-xylulose-5-phosphate reductoisomerase (DXR) of *Centella asiatica*. 3 Biotech.

[16] (2021). Molecular cloning and functional analysis of 1-deoxy-D-xylulose 5-phosphate reductoisomerase from *Santalum album*. Genes.

[17] (2004). [Cloning and analysis of cDNA encoding key enzyme gene (dxr) of the non-MVA pathway in Taxus chinensis cells]. Sheng Wu Gong Cheng Xue Bao.

[18] (2017). Effects of different doses of cadmium on secondary metabolites and gene expression in *Artemisia annua* L. Frontiers of Medicine.

[19] (2010). Computational analysis of the evolution of 1-deoxy-D-xylulose-5-phosphate reductoisomerase, an important enzyme in plant terpene biosynthesis. Chemistry Biodiversity.

[20] (2014). Engineering the MEP pathway enhanced ajmalicine biosynthesis. Biotechnology and Applied Biochemistry.

[21] (2022). Oligochitosan fortifies antioxidative and photosynthetic metabolism and enhances secondary metabolite accumulation in arsenic-stressed peppermint. Frontiers in Plant Science.

[22] (2017). Transcriptome sequencing analysis reveals a difference in monoterpene biosynthesis between scented *Lilium* 'Siberia' and unscented *Lilium* 'novano'. Frontiers in Plant Science.

[23] (2017). Tissue-specific gene-expression patterns of genes associated with thymol/carvacrol biosynthesis in thyme (*Thymus vulgaris* L.) and their differential changes upon treatment with abiotic elicitors. Plant Physiology and Biochemistry.

[24] (2019). Evaluation, characterization, expression profiling, and functional analysis of *DXS *and *DXR* genes of *Populus trichocarpa*. Plant Physiology and Biochemistry.

[25] (2014). Deoxyxylulose 5-phosphate reductoisomerase is not a rate-determining enzyme for essential oil production in spike lavender. Journal of Plant Physiology.

[26] (2020). The organ-specific differential roles of rice DXS and DXR, the first two enzymes of the MEP pathway, in carotenoid metabolism in *Oryza sativa* leaves and seeds. BMC Plant Biology.

[27] (2020). Citral and linalool nanoemulsions: impact of synergism and ripening inhibitors on the stability and antibacterial activity against *Listeria monocytogenes*. Journal of Food Science and Technology.

[28] (2022). Inhibition of *Cronobacter sakazakii* by *Litsea cubeba* essential oil and the antibacterial mechanism. Food.

[29] (2022). Role of *Litsea cubeba* essential oil in agricultural products safety: antioxidant and antimicrobial applications. Plants.

[30] (1974). Effect of vitamin A and Citral on peritoneal adhesion formation. The Journal of Surgical Research.

[31] (2021). Plant-derived bioactive compounds produced by *Streptomyces variabilis* LCP18 associated with *Litsea cubeba* (Lour.) Pers as potential target to combat human pathogenic bacteria and human cancer cell lines. Brazilian Journal of Microbiology.

[32] (2021). Unraveling the anti-bacterial mechanism of *Litsea cubeba* essential oil against *E. coli* O157: H7 and its application in vegetable juices. International Journal of Food Microbiology.

[33] (2012). Chemical composition of essential oils of *Litsea cubeba* harvested from its distribution areas in China. Molecules.

[34] (2022). LcERF19, an AP2/ERF transcription factor from *Litsea cubeba*, positively regulates geranial and neral biosynthesis. Horticulture Research.

[35] (2022). Phytohormone and transcriptome of pericarp reveals jasmonate and *LcMYC2* are involved in neral and geranial biosynthesis in *Litsea cubeba*. Industrial Crops and Products.

[36] (2020). Overexpression of the 3-hydroxy-3-methylglutaryl-CoA synthase gene *LcHMGS* effectively increases the yield of monoterpenes and sesquiterpenes. Tree Physiology.

[37] (2020). Overexpression of geranyl diphosphate synthase small subunit 1 (LcGPPS. SSU1) enhances the monoterpene content and biomass. Industrial Crops and Products.

[38] (2013). Identification of appropriate reference genes for normalizing transcript expression by quantitative real-time PCR in *Litsea cubeba*. Molecular Genetics and Genomics.

[39] (1987). Design and construction of a versatile system for the expression of foreign genes in plants. Gene.

[40] (2016). Digital gene expression profiling to explore differentially expressed genes associated with terpenoid biosynthesis during fruit development in *Litsea cubeba*. Molecules.

[41] (2013). Transcriptome sequencing and expression analysis of terpenoid biosynthesis genes in *Litsea cubeba*. PLoS One.

[42] (2019). High yield of bioactive abietane diterpenes in Salvia sclarea hairy roots by overexpressing cyanobacterial *DXS* or *DXR* genes. Planta Medica.

[43] (2010). Stress and developmental responses of terpenoid biosynthetic genes in *Cistus creticus* subsp. *creticus*. Plant Cell Reports.

[44] (2013). Cloning and characterization of 2-C-methyl-D-erythritol-4-phosphate pathway genes for isoprenoid biosynthesis from Indian ginseng, *Withania somnifera*. Protoplasma.

[45] (2013). Enzyme inhibitor studies reveal complex control of methyl-D-erythritol 4-phosphate (MEP) pathway enzyme expression in *Catharanthus roseus*. PLoS One.

[46] (2022). Overexpression of geranyl diphosphate synthase (*PmGPPS1*) boosts monoterpene and diterpene production involved in the response to pine wood nematode invasion. Tree Physiology.

[47] (2017). Co-expression of peppermint geranyl diphosphate synthase small subunit enhances monoterpene production in transgenic tobacco plants. The New Phytologist.

[48] (2004). Co-expression of three MEP pathway genes and *geraniol 10*-*hydroxylase* in internal phloem parenchyma of *Catharanthus roseus* implicates multicellular translocation of intermediates during the biosynthesis of monoterpene indole alkaloids and isoprenoid-derived primary metabolites. The Plant Journal.

[49] (2000). Functional involvement of a deoxy-D-xylulose 5-phosphate reductoisomerase gene harboring locus of *Synechococcus leopoliensis* in isoprenoid biosynthesis. FEBS Letters.

[50] (2018). Overexpression of *LiDXS* and *LiDXR* from lily (*Lilium* 'Siberia') enhances the terpenoid content in tobacco flowers. Frontiers in Plant Science.

[51] (2022). Rational design of geranylgeranyl diphosphate synthase enhances carotenoid production and improves photosynthetic efficiency in *Nicotiana tabacum*. Science Bulletin.

[52] (2016). Differential contribution of the first two enzymes of the MEP pathway to the supply of metabolic precursors for carotenoid and chlorophyll biosynthesis in carrot (*Daucus carota*). Frontiers in Plant Science.

[53] (2022). Isoprenoid biosynthesis regulation in poplars by methylerythritol phosphate and mevalonic acid pathways. Frontiers in Plant Science.

[54] (2017). Dynamics of monoterpene formation in spike lavender plants. Metabolites.

[55] (2015). Metabolic cross-talk between pathways of terpenoid backbone biosynthesis in spike lavender. Plant Physiology and Biochemistry.

[56] (2018). Improved fruit α-tocopherol, carotenoid, squalene and phytosterol contents through manipulation of *Brassica juncea* 3-HYDROXY-3-METHYLGLUTARYL-COA SYNTHASE1 in transgenic tomato. Plant Biotechnology Journal.

[57] (2015). Mathematical modelling of the diurnal regulation of the MEP pathway in *Arabidopsis*. New Phytologist.

[58] (2010). The isogene *1-deoxy-D-xylulose 5-phosphate synthase 2* controls isoprenoid profiles, precursor pathway allocation, and density of tomato trichomes. Molecular Plant.

