# Supplementary material for: Overexpression of 1-deoxy-D-xylulose-5-phosphate reductoisomerase enhances the monoterpene content in Litsea cubeba
Source: For Res (Fayettev). 2023 Apr 24;3:11. doi: 10.48130/FR-2023-0011 (PMC11524321; doi:10.48130/FR-2023-0011)
Supplement: Supplementary file 1 — Supplementary data to this article can be found online. [file FR-2023-0011-S1.zip › 10.48130_FR-2023-0011-Suppl-TableS1.docx]

**Table S1 Primer list**

| **Purpose** | **Primer name** | **Primer sequence (5′-3′)** |
| --- | --- | --- |
| qRT-PCR | UBC-F | TGTGTGTGTGTGTGTGTGTCC |
|  | UBC-R | CCTTTCTCCACGGTCTTCAA |
|  | LcDXR-RT-F | TCCCCCCTGCACGTAGAC |
|  | LcDXR-RT-R | GCTTCCTTTGCTGGAATCCA |
|  | LcDXS-RT-F | GAAGGATCGATTGGCGGTT |
|  | LcDXS-RT-R | AGCCCATTCAAGGCCAAGA |
|  | LcHMGS1-RT-F | AGGATTGCATGGCTTTCTGT |
|  | LcHMGS1-RT-R | TGGATTTGCTCTTGTCGATG |
|  | LcGPPS.SSU1-RT-F | CGCTGAATTTGCTGTCACC |
|  | LcGPPS.SSU1-RT-R | GAGGAGGTGCCTGAACTGAA |
|  | LcTPS42-qRT-F | GTTGTCCTCAGCGGCTTCTT |
|  | LcTPS42-qRT-R | GCTTGGATCGAATGGAGCAT |
|  | NbDXR-RT-F | CGCTGAATTTGCTGTCACC |
|  | NbDXR-RT-R | TTCAGTTCAGGCACCTCCTC |
|  | NbDXS-RT-F | TGAACAGGACAGTGGCTTCA |
|  | NbDXS-RT-R | AACCCCACGTGACCTTTTCT |
|  | NbHMGR-RT-F | AAGGTGTACAAAACGTTCTGGATTA |
|  | NbHMGR-RT-R | ACTTTCTTCACCACTTCTTCCTTG |
|  | NbHMGS-RT-F | CCGCCTACTTGCCTCCGA |
|  | NbHMGS-RT-R | CTCGAGACGACCAATTTGCTTA |
|  | NbGPPS-RT-F | GGCTTGGCATTTCAATTAATAGAC |
|  | NbGPPS-RT-R | ATCAAATCCTCGGTCCACCA |
|  | Actin-F | TCCTGATGGGCAAGTGATTAC |
|  | Actin-R | GAATCCACGAGACCACATACAA |
| Overexpression | LcDXR-1300-F | tcagcagtcgaagagcATGGCTTTGAAATCCCCCCTG |
|  | LcDXR-1300-R | ttagcgtgtgaagagcAGCCAGGACGGGTCTTTTC |
| Clone | LcDXR-F | ATGGCTTTGAAATCCCCCCTGCAC |
|  | LcDXR-R | TTAAGCCAGGACGGGTCTTTTCCCAG |
| Enzymatic assays | LcDXR-F | ATGGCTGATATCGGATCCATGGCTTTGAAATCCCCCCTG |
|  | LcDXR-R | GAGTGCGGCCGCAAGCTTTCAAGCCAGGACGGGTCTTTTC |
