# Supplementary material for: Overexpression of 1-deoxy-D-xylulose-5-phosphate reductoisomerase enhances the monoterpene content in Litsea cubeba
Source: For Res (Fayettev). 2023 Apr 24;3:11. doi: 10.48130/FR-2023-0011 (PMC11524321; doi:10.48130/FR-2023-0011)
Supplement: Supplementary file 1 — Supplementary data to this article can be found online. [file FR-2023-0011-S1.zip › 10.48130_FR-2023-0011-Suppl-FigureS1.docx]

**Supporting Information**

**Fig. S1** Promoter sequence of *LcDXR*.
